# The Assessment of Functional Movement in Children and Adolescents: A Systematic Review and Meta-Analysis

**DOI:** 10.1007/s40279-021-01529-3

**Published:** 2021-09-15

**Authors:** Wesley O’Brien, Zeinab Khodaverdi, Lisa Bolger, Giampiero Tarantino, Conor Philpott, Ross D. Neville

**Affiliations:** 1 School of Education, Sports Studies and Physical Education Programme, 2 Lucan Place, Western Road, University College Cork, Cork, Ireland; 2 Department of Motor Behaviour, Faculty of Physical Education and Sport Sciences, Kharazmi University, Tehran, Iran; 3 Department of Sport, Leisure and Childhood Studies, Cork Institute of Technology, Cork, Ireland; 4 School of Public Health, Physiotherapy and Sports Science, University College Dublin, Dublin, Ireland

## Abstract

**Background:**

The Functional Movement Screen™ (FMS™) is an assessment of human movement that may signal potential deficits that could predispose an otherwise healthy person to injury risk. FMS™ scores are well reported in both athletic and adult samples. However, to date, there has been no comprehensive systematic review and meta-analysis of FMS™ data among school-aged children and adolescents.

**Objective:**

We aimed to systematically review and analyse functional movement proficiency of children and adolescents, specifically when assessed using the FMS™, and to establish initial normative values for the FMS™ in this population group and to further estimate differences in functional movement proficiency between the sexes, by school level (i.e., between primary and secondary school-level children and adolescents), and based on differences in child and adolescent body mass index (BMI).

**Methods:**

In accordance with the Preferred Reporting Items for Systematic Reviews and Meta-Analyses (PRISMA) guidelines, prospective studies were identified from searches across eight databases (MEDLINE, SPORTDiscus, CINAHL, Web of Science, EMBASE, ERIC, PsychINFO and PubMed), without any date restrictions, up to December 2020. The primary meta-analysis estimated the overall FMS™ score for school-aged children and adolescents across published studies. An additional three subgroup meta-analyses estimated comparisons for FMS™ data with school level, sex, and BMI across published studies. FMS™ data were meta-analysed using a number of different meta packages (Schwarzer et al. in Meta-Analysis with R, 1st ed, Springer International Publishing, Berlin, 2015), available in R Studio.

**Results:**

A total of 19 articles were included in the systematic review. Meta-analysis revealed a weighted FMS™ mean score of 14.06, with a standardised *Tau* value of 0.56, signalling a moderate-to-large degree of variability in FMS™ means between studies. The difference in FMS™ means between samples of males (weighted FMS™ mean 13.91) and females (weighted FMS™ mean 14.56) was compatible with a possible small effect size (standardised mean difference − 0.27). The variability in FMS™ means between studies was approximately five times greater in samples of secondary school children (factor difference in *Tau* values 5.16). The final meta-regression identified a negative association between BMI and FMS™ scores (*r* =  − 0.42), which signalled a moderate-to-large difference in FMS™ scores between healthy weight and overweight children/adolescents.

**Conclusion:**

This systematic review and meta-analysis represents a novel and important synthesis of published FMS™ data from groups of children and adolescents. The study signals possible sex- and age-related differences in FMS™ scores, as well as a clear negative relationship between BMI and functional movement proficiency. More longitudinal research is needed to better understand the developmental trajectory and the effects of maturation milestones on FMS™ proficiency. Additional research is also needed to identify the types of interventions that could improve functional movement proficiency among ‘at risk’ groups, who are susceptible to functional movement deficiency, and whether changes in body composition mediate the relationship between these interventions and the improvement of FMS™ scores.

## Key Points

**Table:** 

Functional movement proficiency, including postural control, stability, flexibility, neuromuscular coordination, and balance, represents an important building block for lifelong engagement and potentially injury-free-engagement in organised sport.
The Functional Movement Screen™ (FMS™) represents the pre-eminent assessment tool for evaluating functional movement; however, to date, research has been primarily focused on FMS™ in the context of athletic populations.
This study is the first to synthesise published FMS™ data from samples of children and adolescents, thereby providing normative reference values for practitioners working in physical activity, physical education, and sport settings.
Possible sex- and age-related differences in FMS™ scores are evident in children and adolescent samples. There is also a clear negative relationship between body mass index and functional movement proficiency in this population group.
Further longitudinal research is needed to better understand the developmental trajectory and the effects of maturational milestones on FMS™ proficiency in children and adolescents.

## Introduction

Functional movement refers to movement of the body that is characterised by adequate joint and muscle function, and by a movement efficiency that has been shown to minimise the risk of injury [2, 3]. Functional movement has been theorised as a precursor for higher-order, or more complex, forms of bodily movement [4], and assessments typically involve the measurement of postural control motion, stability, flexibility, neuromuscular coordination, and balance [2, 3, 5]. These subcomponents of functional movement have not only been recognised as enablers of high-quality bodily movement but there is also research evidence to show that these subcomponents of functional movement predict important markers of health [6–8].

The Functional Movement Screen™ (FMS™) is the pre-eminent screening tool for assessing functional movement, with a large body of empirical research conducted and synthesised to date on youth athletic populations as well as adult populations [2, 9, 10]. FMS™ is comprised of seven different movements designed to evaluate mobility, flexion, extension and stability [2, 3]. These seven movements and their measurement purpose comprise (1) active straight-leg raise (evaluates hamstring flexibility, core stability, and active hip mobility); (2) trunk stability push-up (core and spinal stability, some upper body strength); (3) shoulder mobility (range of motion of the shoulders and internal and external rotation of both shoulders); (4) deep squat (mobility and stability of the hips, knees, ankles and thoracic spine); (5) rotary stability (neuromuscular coordination and stability throughout the shoulders and spine); (6) hurdle step (bilateral mobility of hips, knees and ankles in a single-leg stance); and (7) the in-line lunge (hip, knee ankle mobility and spinal stability) [2, 3]. The individual FMS™ items are scored on a 0–3 ordinal scale (with 3 being the optimal score), with five of the seven movements scored bilaterally (i.e., both left and right side of the body) and with the lower value on the left or right side contributing to the overall, or composite, score [2, 3]. The maximum overall composite FMS™ score across all seven movement assessments is 21 [2, 3].

Impaired functional movement as screened by lower scores on the FMS™ has been linked to a higher level or risk for injury [11, 12]. Previous studies and systematic reviews have suggested that a composite score below 14 could be a marker of an increased risk of acute and chronic injuries [11–13]. Despite caution being applied to this risk threshold within the literature [12, 14, 15], there is some established consensus that lower FMS™ scores are indicative of poor movement competencies and are hence worth monitoring [12, 16–18]. For example, several FMS™ movements evaluate thoracic mobility, namely the deep squat, in-line lunge, and shoulder mobility exercise [2, 3, 19], and a higher level of thoracic mobility may account for the biomechanics required to perform locomotor skills [17]. Moreover, numerous studies have found inverse associations between FMS™ scores and agility (i.e., as FMS™ scores increase, agility tests are performed with greater speed, reducing the time spent in activity), repeated sprint ability (higher FMS™ scores correlating with lower mean time sprint scores), and short-dose anaerobic tasks [20–22]. This suggests that functional movement positively influences running outcome capacities [20–22]. Greater functional movement competency has been linked with improved levels of static and dynamic balance in both adolescent and young adult populations [23, 24]. Therefore, functional movement competency and the assessment of such movement patterns could prove foundational to lifelong physical activity (PA) by providing the stability and neuromuscular control that are deemed essential to all forms of movement and exercise [6, 21, 25].

While previous studies have been conducted on the utility of the FMS™ as a tool for detecting injury, as well as the tool’s sensitivity to predicting athletic performance [11, 20, 23, 24, 26], to date there has been no attempt by the sport science research community to synthesise FMS™ in typically developing children and adolescent groups (despite the tool’s suggested value as an assessment tool in this population group) [11, 15, 26]. The primary aim of this paper is therefore to systematically review and meta-analyse the available published international FMS™ data among typically developing children and adolescents using the 0–21 scoring scale. With the exception of one publication, no empirical research outputs on the 100-point scale [27] were found among typically developing children and adolescent groups during the systematic review search. Our primary aim was to establish initial normative values for FMS™ in this population group for the first time, which will aid physical education as well as other recreational activities and school sports. Our secondary aim was to explore the extent to which these normative values differed between the sexes and by school level (i.e., between primary and secondary school cohorts) and to estimate whether FMS™ scores differed significantly for children and adolescents by levels of body mass index (BMI). Independent of age and sex associations for children and youth, few anthropometric correlates have been reported across FMS™ studies, with the exception of BMI. BMI is an index for one’s weight, relative to their height [28]. BMI is commonly used in adult and childhood research in relation to PA and health, given its validity as an indirect measure of adiposity and weight status [28, 29]. We believe such a research synthesis exploration of correlates will serve to build up a robust body of research evidence that can be used to develop effective strategies for enhancing functional movement among all school-aged children, irrespective of their level of engagement within competitive sport.

## Methods

A systematic search was conducted in accordance with the Preferred Reporting Items for Systematic Reviews and Meta-Analyses (PRISMA) guidelines [30].

### Search Strategy

Eight databases, MEDLINE, SPORTDiscus, CINAHL, Web of Science, EMBASE, ERIC, PsychINFO and PubMed, were searched without any date restrictions, up to December 2020, for articles relating to the FMS™ assessment in typically developing school-aged children and adolescents. The authorship team also searched the reference lists of relevant articles and review articles to identify any other studies that might have been missed during the electronic database search. The main search group terms in the first seven databases were: (adolescent* OR pupil* OR student* OR youth* OR child* OR teenager*) AND (school* OR classroom* OR physical education* OR PE*) AND (functional movement skills* OR functional movement screen*). The research strategy in PubMed was to use Medical Subject Heading (MeSH) terms. MeSH is the National Library of Medicine’s (NLM’s) controlled vocabulary or subject heading list and reflects subject content of journal articles as they are published [31]. The main search group MeSH terms in PubMed were: (adolescent [MeSH Terms] OR pupil* OR student* OR youth [MeSH Terms] OR children* [MeSH Terms] OR teenager* [MeSH Terms]) AND (school* [MeSH Terms] OR classroom* OR physical education* OR PE* [MeSH Terms]) AND (functional movement skills* OR functional movement screen*).

### Eligibility Criteria

The population samples in the identified studies under review included typically developing school-aged children and adolescents (< 18 years of age) from varied socioeconomic backgrounds.

A qualitative synthesis was performed on all the studies in this review. The following criteria were used for the inclusion of a study in this review: (1) the study must have a full assessment of the established FMS™ tool [2, 3, 32], according to the 0–21 points scoring system; and (2) only articles published in English and in peer-reviewed journals were considered. Books, reviews, abstracts, commentaries, qualitative studies and case studies were excluded.

### Data Extraction

Two authors (LB, CP) independently extracted data in a Microsoft Excel spreadsheet (Microsoft Corporation, Redmond, WA, USA), which included authors, year of publication, country in which the study was conducted, sample characteristics (size, age and sex), children’s school level (primary, secondary), and study design. Any discrepancies found were discussed between the authors until agreement was reached. Additional data for the meta-analyses (FMS™ means and standard deviations [SDs]) were extracted from the articles included in this systematic review. If a study did not report enough data, the study’s authors were emailed for the raw data.

### Criteria for Risk-of-Bias Assessment

Three authors (ZK, WOB, LB) independently assessed the risk of bias in the included studies. The criteria for assessing the risk of bias in the studies were adapted from the Strengthening the Reporting of Observation Studies in Epidemiology (STROBE) statement. The criteria identified as relevant to the current review have previously been used in a review of a similar area [33] and include the following.

Were the participants likely to be representative of the population (i.e., country, state/region level)? Were schools or students randomly selected or were other data provided to indicate population representativeness?
Of those who consented to the study, did an adequate proportion have complete data for the outcome and all correlates of interest (i.e., no more than 20% of data were missing from a cross-sectional study and no more than 30% for a longitudinal study)?
(a) Did the study report the sources and details of functional movement assessment and were valid measures of functional movement used (validation in same age group published or validation data provided in the manuscript)? (b) Did the functional movement screen tool used report adequate reliability of functional movement assessment?
Did the study report the sources and details of assessment of potential correlates? Each item on the scale was individually considered and coded as either ‘yes’ (tick) or ‘no’ (X). Following the review process, articles in which disagreements were found were further reviewed by the group of six authors, and consensus was reached following discussion.

### Screening

Two disciplinary specific reviewers (ZK, LB) with systematic review experience worked independently to screen the title and abstract of the studies for relevance to the review. Authors searched relevant full-text articles and evaluated the studies for inclusion considerations, according to the aforementioned criteria. The reference lists of the included studies were also reviewed for potential papers. In instances of disagreements over the inclusion of a certain study or the specific data obtained, the matter was resolved through discussion with a third disciplinary specific reviewer (WOB).

### Statistical Analyses

Sixteen studies provided appropriate data for inclusion in a meta-analysis. Four independent meta-analyses were performed. The purpose of the first meta-analysis was to estimate the overall FMS™ score for school-aged children across published studies, while the second and third meta-analyses were subgroup meta-analyses that investigated the differences in these FMS™ means between primary and secondary school children, and between males and females, respectively. The final meta-analysis provided a pooled estimate of the association between BMI and FMS™ scores across published studies.

FMS™ data were meta-analysed using a number of different meta packages [1], available in R Studio [34]. For the first three meta-analyses, raw FMS™ means were used. The FMS™ mean score for each study was entered into the model, alongside its respective standard error. The weighting factor for the meta-analyses was the inverse of this standard error [35]. For the first meta-analysis estimating the overall FMS™ mean for school-aged children, data were pooled using the metagen function, and a random effects model was specifically based on the Hartung–Knapp–Sidik–Jonkman adjustment (a method shown to robustly estimate between-study variance when the number of studies in the meta-analysis is small and when there is evidence of substantial between-study heterogeneity) [36]. To estimate differences in FMS™ means between children at different school levels (primary and secondary) and between male and female children, school-level and sex of the child were coded as binary categorical variables. A subgroup meta-analysis function was subsequently applied using the dmetar package and the source code extensions for the metagen package provided by Harrer et al. [34]. This subgroup meta-analysis resulted in an estimate of both the FMS™ mean for each group and of the standardised difference in FMS™ means between groups. These standardised differences were calculated by dividing the raw mean difference between groups by the between-subject SD [37]. Magnitude thresholds for evaluating the effect size of these standardised differences were based on the following scale: < 0.2 = trivial; 0.2–0.6 = small; 0.6–1.2 = moderate; 1.2–2.0 = large; and > 2.0 = very large [37]. For the final meta-analysis, correlation coefficients representing the association between BMI and FMS™ scores from eight published studies were pooled using the metacor function, with a random effects model specified and a weighting factor based on the Fisher *r*-to-*Z* transformation [34]. Magnitude thresholds for evaluating the effect size of these correlations were based on the following scale: < 0.1 = trivial; 0.1–0.3 = small; 0.3–0.5 = moderate; 0.5–0.7 = large; and > 0.7 = very large [37].

Uncertainty in the pooled estimate for each of the four meta-analyses is presented as a 95% confidence interval (CI). Between-study heterogeneity is evaluated using the *I*^2^ statistic, which represents the proportion of variability in study effect sizes that is not caused by sampling error. The thresholds reported by Higgins et al. [38] were used to interpret the magnitude of *I*^2^: < 40%, trivial; 30–60%, moderate; 50–90% substantial; and 75–100%, considerable. Between-study variability was also estimated using the *Tau*^2^ statistic. The magnitude of this *Tau*^2^ statistic was assessed by calculating its square root, resulting in a value that represents the effective between-study SD (i.e., *Tau*). Magnitude thresholds for evaluating the effects size of these SDs were (following the suggestion of Hopkins [39]) based on the following scale: < 0.1 = trivial; 0.1–0.3 = small; 0.3–0.6 = moderate; 0.6–1.2 = large; and > 1.2 = very large [37]. Magnitude thresholds for evaluating the difference in these SDs were based on logarithmic transformation of the SDs expressed as factors. Magnitude thresholds for evaluating factor differences in these SDs were: < 1.12 = trivial; 1.12–1.41, small; 1.41–2.0, moderate; 2.0–3.2, large; and > 3.2, very large [37].

## Results

**Fig. 1:**
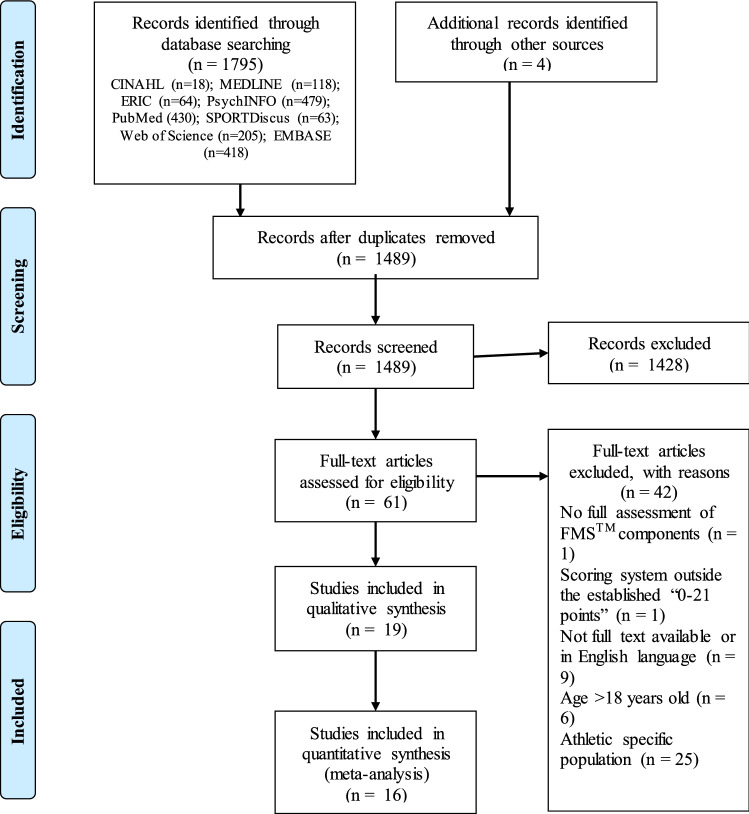
Studies included in this systematic review. *FMS™* Functional Movement Screen™

The search strategy identified 1799 potentially relevant articles, of which 310 were removed as duplicates. Of the remaining 1489 articles, 1428 were excluded because they did not meet the inclusion criteria. Following screening and detailed assessment, a further 42 articles were excluded for different reasons (no full FMS™ means provided, different scoring system, full text unavailable or language different than English, not school-aged population, and athletic population), resulting in 19 studies being deemed suitable for this systematic review (Fig. 1).

### Overview of Studies

In terms of the 19 studies identified (Fig. 1), all were published between 2012 and 2020. One of the authorship team extracted the descriptive and demographic data from the studies (ZK), and these data were confirmed and checked by the principal investigator (WOB). The included studies reflected a range of participant ages within the primary and secondary school age groups (9–18 years), with four studies not specifying the school stage of the participants [40–43]. Of the remaining 13 studies that specified the school stage of the participants, 7 studies were undertaken with secondary school-aged children [44–50] and 6 studies were undertaken with primary school-aged children [4, 51–55].

Across the 19 studies, 5 were conducted in Spain [41, 49, 52, 53, 55], 3 in the US [42, 43, 45], 3 in Ireland [46–48], 3 in the UK [4, 50, 51], 2 in Croatia [56, 57] and a single study each from India [44], Iran [40] and Moldova [54]. Fourteen of the study designs were cross-sectional [4, 40, 44, 46–49, 51–57], and a single study each was a cluster randomised controlled trial [45], randomised controlled trial [43], quasi-experimental [41], matched pairs experiment [50], and a single-arm trial [42]. The sample sizes ranged from 20 [42] to > 1000 [44]. Approximately one-third of the studies (*n* = 6) had samples of more than 300 (Table 1).

**Table 1:** Characteristics of the included studies

Study	Year	Country	*N*	Sex (B, G)	Age, years^a^	School stage	Design	Live/retrospective FMS™ scoring	Training level of rater(s)
Abraham et al. [44]	2015	India	1005	548, 457	10–17	Secondary	CS	Unclear	Trained
Coker [45]	2018	USA	120	54, 66	13.2 ± 0.4	Secondary	CRCT	R	Trained
Duncan and Stanley [51]	2012	UK	58	29, 29	10.7 ± 0.4	Primary	CS	Unclear	Trained
Duncan et al. [4]	2013	UK	90	38, 52	9.6 ± 1.4	Primary	CS	Unclear	Trained
García-Jaén et al. [55]	2018	Spain	40	20, 20	8.5 ± 0.5	Primary	CS	R	Trained
García-Pinillos et al. [53]	2019	Spain	172	89, 83	9.7 ± 1.6	Primary	CS	Unclear	Trained
García-Pinillos et al. [52]	2018	Spain	333	164, 169	9.7 ± 1.5	Primary	CS	Unclear	Trained
Ghasempoor et al. [40]	2018	Iran	700	350, 350	9–18	Not specified	CS	Unclear	Trained
Karuc et al. [56]	2020	Croatia	652	331, 321	16–17	Secondary	CS	Unclear	Trained
Karuc et al. [57]	2020	Croatia	730	362, 368	16–17	Secondary	CS	Unclear	Trained
Lester et al. [46]	2017	Ireland	181	108, 73	14.4 ± 1.0	Secondary	CS	R	Trained
Mitchell et al. [54]	2015	Moldova	77	39, 38	9.3 ± 0.1	Primary	CS	R	NR
Molina-Garcia et al. [41]	2019	Spain	56	23, 33	8–12	Not specified	QE	R	FMS™ Certified
Nourse et al. [42]	2015	USA	20	11, 9	14.5 ± 2.1	Not specified	SAT	Unclear	NR
O'Brien et al. [47]	2018	Ireland	219	120, 99	14.5 ± 1.0	Secondary	CS	R	Trained
Philpott et al. [48]	2020	Ireland	373	195, 178	12–16	Secondary	CS	R	Trained
St. Laurent et al. [43]	2018	USA	28	13,15	9.3 ± 1.5	Not specified	RCT	Unclear	Trained
Vernetta-Santana et al. [49]	2020	Spain	35	11, 24	12.2 ± 0.4	Secondary	CS	R	Trained
Wright et al. [50]	2015	UK	22	NR	13.4 ± 1.0	Secondary	MPE	R	NR

*CRCT* cluster randomised controlled trial, *CS* cross-sectional, *MPE* matched pairs experiment, *QE* quasi-experiment, *RCT* randomised controlled trial, *SAT* single-arm trial,

*FMS™* Functional Movement Screen™, *R* retrospective, *B* boy, *G* girl, *N* study sample size, *NR* data not reported

^a^Ages were reported in studies either as a range or as mean age ± standard deviation

A total of 13 studies (70%) investigated two (7 studies) [4, 46–49, 51, 56] or more (6 studies) [41, 43, 44, 52–54] correlates of FMS™. The commonly investigated correlates among studies were biological and demographic correlates, such as sex (14 studies) [4, 41, 43–47, 49, 52–57], BMI (9 studies) [4, 41, 43, 49, 51–54, 56] and school level (7 studies) [43, 44, 46, 48, 52–54]. A total of 6 studies assessed other correlates such as maturity, core strength, perceived functional movement competence, previous injury, postural angel, fitness indicators, fitness components, physical self-confidence and PA level.

### Overview of Studies’ Risk of Bias

The assessment of the study risk of bias is presented in Table 2. Of the 19 included studies, 9 (47%) had samples that were considered representative of the study population [40, 44, 46–48, 52, 53, 56, 57]. All studies had minimal missing data, and all used the FMS™ assessment tool [2, 3], which is reported to be both valid and reliable in the assessment of functional movement among the included samples. The majority of studies (89%; 17/19 studies) examined potential correlates [4, 40, 41, 43–49, 51–57] in a valid and reliable manner.

**Table 2:** Risk-of-bias summary

Study details		Study quality				Correlates assessed				
Study	Year	Representative sampling	Minimal missing data	Valid FMS tool	Reliable FMS tool	Number of correlates	Age	Sex	BMI	Other correlates
Abraham et al. [44]	2015	✔	✔	✔	✔	5	✔	✔	**X**	Height, weight, previous injury during the last 6 months
Coker [45]	2018	**X**	✔	✔	✔	1	**X**	✔	**X**	NA
Duncan et al. [4]	2013	**X**	✔	✔	✔	2	**X**	✔	✔	NA
Duncan and Stanley [51]	2012	**X**	✔	✔	✔	2	**X**	**X**	✔	PA levels (average steps/day)
García-Jaén et al. [55]	2018	**X**	✔	✔	✔	1	**X**	✔	**X**	NA
Garcia-Pinillos et al. [52]	2018	✔	✔	✔	✔	3	✔	✔	✔	NA
Garcia-Pinillos et al. [53]	2019	✔	✔	✔	✔	3	✔	✔	✔	NA
Ghasempoor et al. [40]	2018	✔	✔	✔	✔	1	**X**	**X**	**X**	Maturity (assessed using the predicted maturity offset formula)
Karuc et al. [56]	2020	✔	✔	✔	✔	2	**X**	✔	✔	NA
Karuc et al. [57]	2020	✔	✔	✔	✔	1	**X**	✔	**X**	NA
Lester et al. [46]	2017	✔	✔	✔	✔	2	✔	✔	**X**	NA
Mitchell et al. [54]	2015	**X**	✔	✔	✔	5	✔	✔	✔	Core strength, postural angles
Molina-Garcia et al. [41]	2019	**X**	✔	✔	✔	4	**X**	✔	✔	Fatness indicators (BMI, WC, fat mass index, body fat %), fitness components (upper and lower body muscular strength, cardiorespiratory fitness, speed-agility, VO_2_max)
Nourse et al. [42]	2015	**X**	✔	✔	✔	0	**X**	**X**	**X**	NA
O'Brien et al. [47]	2018	✔	✔	✔	✔	2	**X**	✔	**X**	PSC
Philpott et al. [48]	2020	✔	✔	✔	✔	2	✔	**X**	**X**	Perceived functional movement competence
St. Laurent et al. [43]	2018	**X**	✔	✔	✔	3	✔	✔	✔	NA
Vernetta-Santana et al. [49]	2020	**X**	✔	✔	✔	2	**X**	✔	✔	NA
Wright et al. [50]	2015	**X**	✔	✔	✔	0	**X**	**X**	**X**	NA

*NA* not applicable, *PSC* physical self-confidence, *PA* physical activity, *FMS* Functional Movement Screen, *BMI* body mass index, *WC* waist circumference, *VO*_*2*_*max* maximum aerobic capacity, ✔ indicates ‘yes’, *X* indicates ‘no’

### Meta-Analysis

Two authors (RN, GT) extracted data for the meta-analysis. Four meta-analyses were undertaken to estimate (1) normative values for the FMS™ in typically developing school-aged children and adolescents, differences in these normative values between samples of (2) males and females and (3) children and adolescents at primary and secondary school level, and (4) differences in FMS™ for children and adolescents with below and above average levels of BMI. The outcomes of these four meta-analyses are described in the following subsections. Following Hopkins’ recent recommendations about the use of standardisation to calculate and assess effect magnitudes in meta-analyses, a final complementary meta-analysis was also undertaken to estimate a between-subject SD for FMS™. This SD was necessary to calculate standardised effects representing the difference in FMS™ means between samples of males and females, and between primary and secondary school-aged children and adolescents [i.e., meta-analysis (2) and (3)]. The outcomes of this complementary meta-analysis are not central to the aims of this study and are therefore described in the online-only supplementary material.

#### Normative Values for Functional Movement Screen™ (FMS™) Among Typically Developing Children and Adolescents

**Fig. 2:**
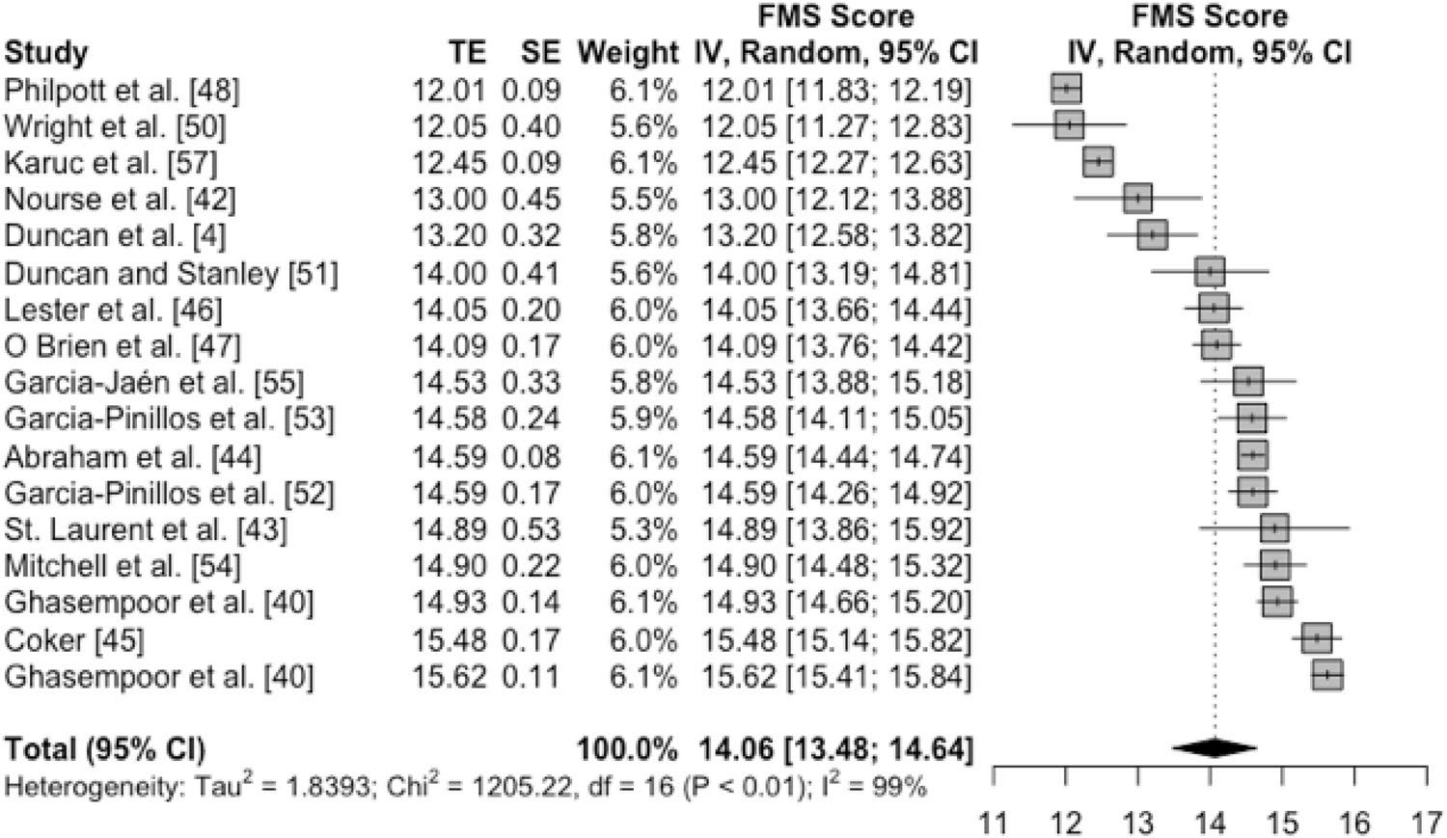
Forest plot of FMS™ means and 95% CIs from samples of typically developing children and adolescents. The vertical dotted line represents the pooled meta-analytic mean. *FMS™* Functional Movement Screen™, *CI* confidence interval, *TE* treatment effect, *SE* standard error, *IV* inverse variance, *df* degrees of freedom

Of the 19 studies that were included in the overall systematic review, 16 studies provided sufficient data for 17 samples that were deemed eligible for inclusion in the meta-analysis to estimate normative values for FMS™. Figure 2 provides descriptive statistics, weighted mean values, and associated uncertainties for FMS™ for each of the 17 samples (left of Fig. 2). These study-level weighted mean values and their respective 95% CIs are also displayed on a forest plot (right) alongside the pooled meta-analytic mean (black diamond). Considerable levels of heterogeneity were observed for the meta-analytic mean (weighted mean FMS™ 14.06, 95% CI 13.48–14.64), with a statistically significant Chi-square and an *I*^2^ value of 99%. The *Tau* value representing the between-study SD in FMS™ means also signalled substantial heterogeneity, with an average difference between study means of 1.36 units of the measure. This average difference between study means, once standardised, was a moderate-to-large effect size (0.56).

#### Difference in FMS™ Means Between Males and Females

**Fig. 3:**
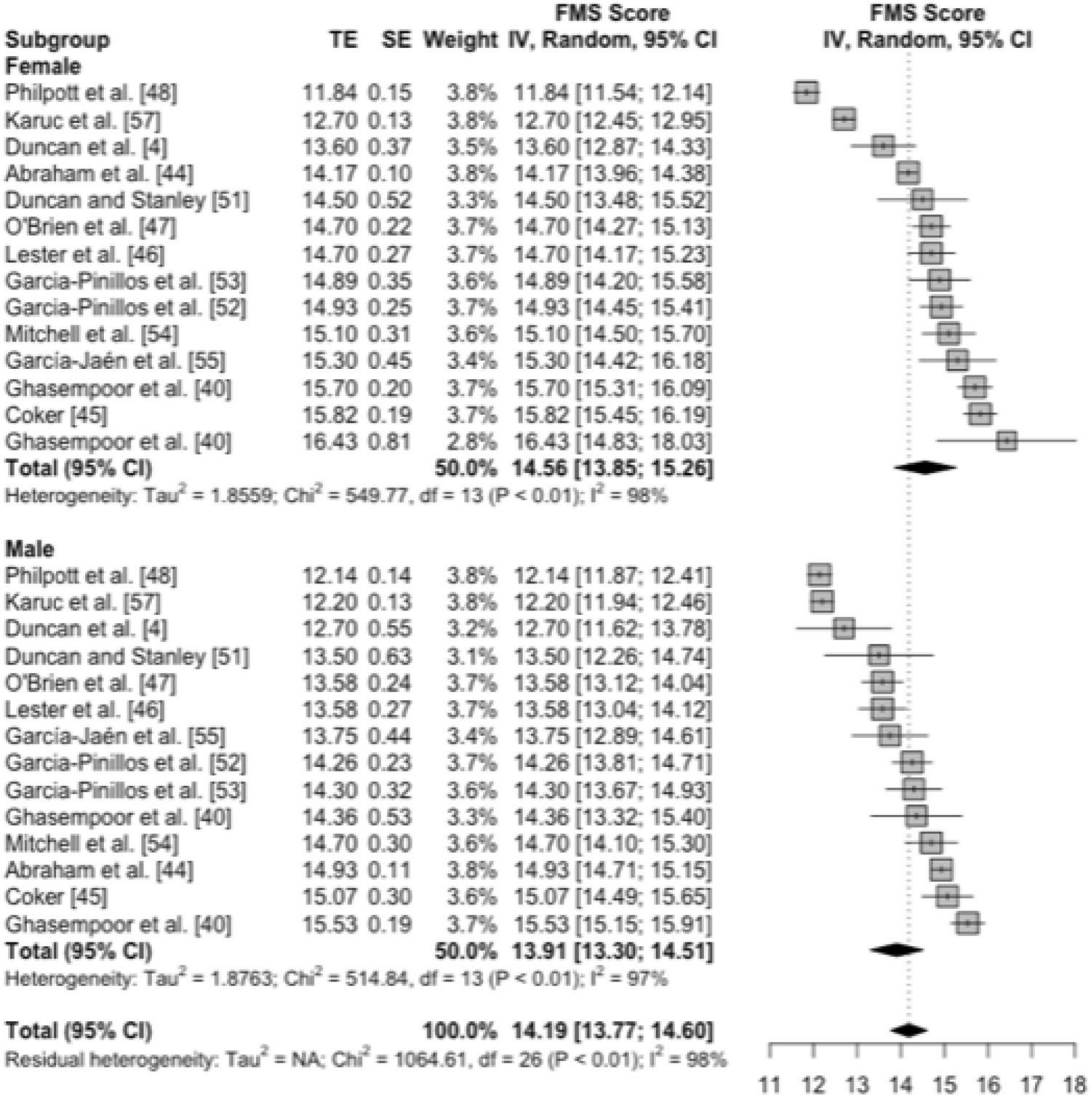
Forest plot of FMS™ means and 95% CIs from samples of typically developing children and adolescents, grouped by sex. The vertical dotted line connecting to the diamond represents the pooled meta-analytic mean, without regard for sex. Floating diamonds represent subgroup meta-analytic means. *FMS™* Functional Movement Screen™, *CI* confidence interval, *TE* treatment effect, *SE* standard error, *IV* inverse variance, *df* degrees of freedom

Of the 16 studies that were eligible for inclusion in the meta-analysis, 13 studies provided descriptive data for 14 independent samples of males and 14 independent samples of females. Figure 3 summarises the outcomes of a subgroup meta-analysis estimating the differences in FMS™ means between these samples of males and females. The effect size representing the difference in FMS™ means between samples of males (weighted mean FMS™ 13.91, 95% CI 13.30–14.51) and females (weighted mean FMS™ 14.56, 95% CI 13.85–15.26) was small in magnitude (standardised mean difference − 0.27, 95% CI − 0.64 to 0.10); however, the effect statistic was not statistically significant (*p* = 0.14) and should therefore be considered as only possibly compatible with a small difference. The subgroup meta-analysis also indicated that differences between samples of males and females did not account for a substantial proportion of the variability in FMS™ means between studies (i.e., a 1% reduction in the *I*^*2*^ value to 97%, and a difference in *Tau* values for samples of males and females of less than 1/10th of a unit of the measure, representing a trivial effect magnitude).

#### Difference in FMS™ Means Between Primary and Secondary School Level

**Fig. 4:**
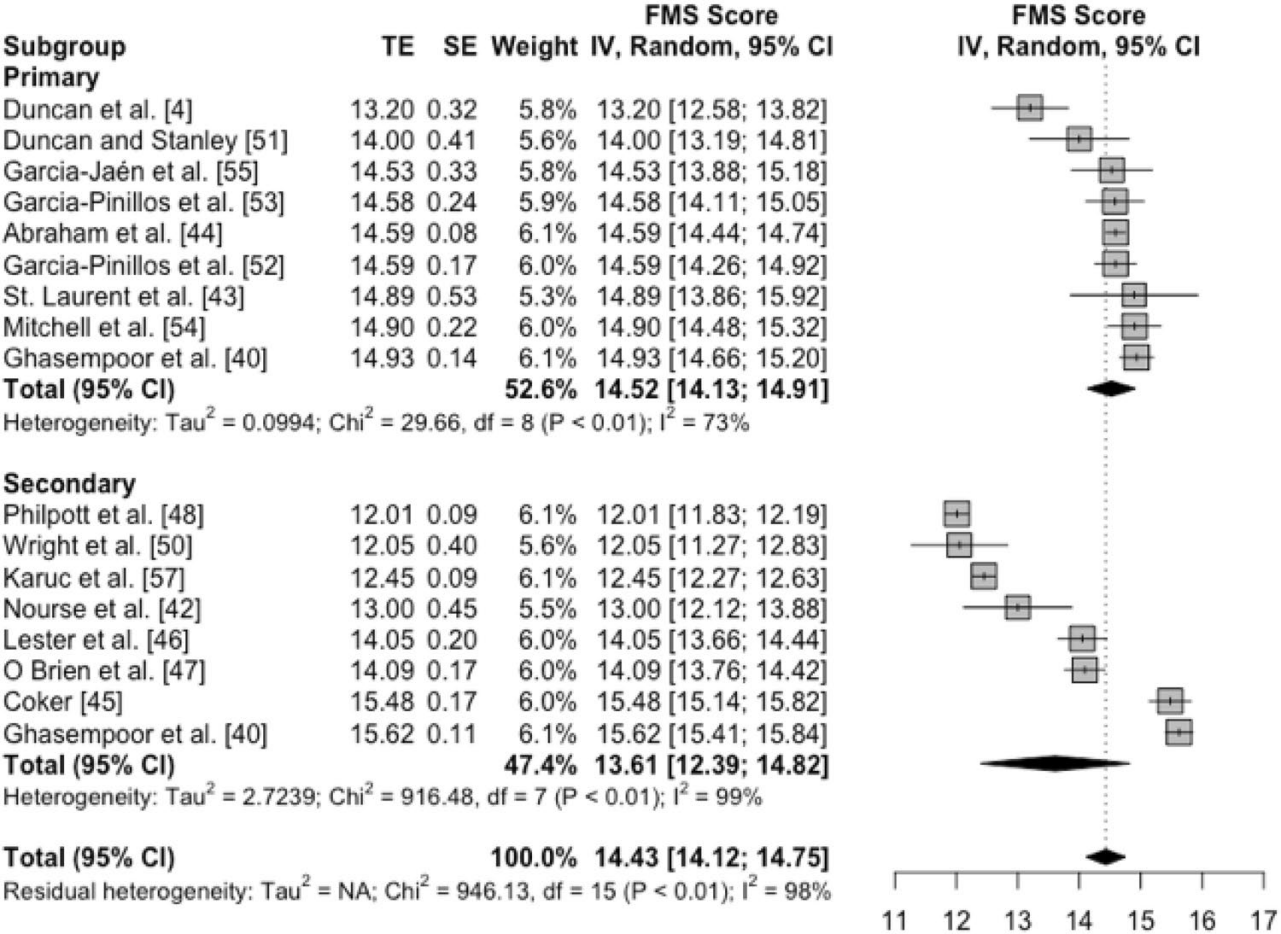
Forest plot of FMS™ means and 95% CIs from samples of typically developing children and adolescents, grouped by school level. The vertical dotted line connecting to the diamond represents the pooled meta-analytic mean, without regard for school level. Floating diamonds represent subgroup meta-analytic means for samples of primary and secondary school-level children. *FMS™* Functional Movement Screen™, *CI* confidence interval, *TE* treatment effect, *SE* standard error, *IV* inverse variance, *df* degrees of freedom

Data for nine independent samples of primary school children and eight independent samples of secondary school children and adolescents were also reported across the 16 studies included in the meta-analysis. Figure 4 summarises the outcomes of a subgroup meta-analysis estimating differences in the pooled means for FMS™ values between primary and secondary school-aged children and adolescents. The effect size of the difference in FMS™ values between primary (weighted mean FMS™ 14.52, 95% CI 14.13–14.91) and secondary (weighted mean FMS™ 13.61, 95% CI 12.39–14.82) school-aged children and adolescents was small in magnitude (standardised mean difference − 0.36, 95% CI − 0.82 to 0.11); however, the difference was not statistically significant (*p* = 0.12). This subgroup meta-analysis indicated that school level accounted for a substantial proportion of the variability in FMS™ values between studies. For example, the *I*^2^ value for primary school-aged children (73%) was substantially lower than the *I*^2^ value for secondary school adolescents (99%). Our post hoc estimate of the difference in *Tau* values between studies of primary (*Tau* = 0.32) and secondary (*Tau* = 1.65) school-aged participants revealed that the variability in FMS™ means was approximately five times greater for samples of secondary school-aged participants (factor difference in *Tau* values 5.16, 95% CI 2.40–11.06). This effect magnitude represents a very large difference (i.e., factor difference > 3.2) in the between-study variability between primary and secondary school-aged children and adolescents.

#### Association Between FMS™ and Body Mass Index

**Fig. 5:**
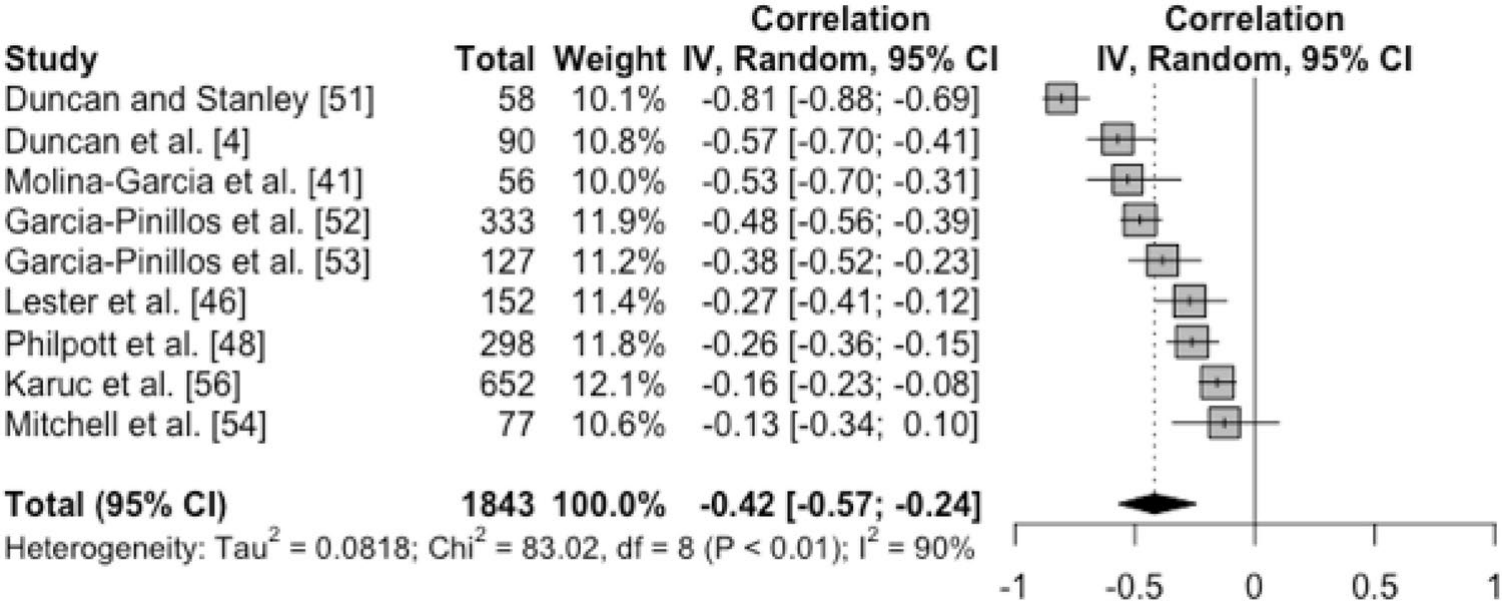
Forest plot of correlation coefficients ± 95% CIs representing the association between FMS™ and BMI score across studies. The vertical line represents a correlation of zero. *FMS™* Functional Movement Screen™, *CI* confidence interval, *BMI* body mass index, *IV* inverse variance, *df* degrees of freedom

Of the 19 studies that were included in the systematic review, 9 studies provided correlational data with which we could meta-analyse the association between FMS™ for children and adolescent levels of BMI. Figure 5 summarises the outcomes of this final meta-analysis. The pooled correlation coefficient (*r*) was negative and compatible with a moderate-to-large effect size (*r* =  − 0.42, 95% CI − 0.57 to − 0.24). This means that, on average, when subjects’ BMI values increased by 1 SD, their FMS™ score decreased by approximately 0.4 of an SD. From these data, we were also able to estimate the differences in FMS™ scores between children within the range of healthy weight (between the 25th and 50th percentiles) and overweight (between the 90th and 95th percentile). The resulting difference indicated that FMS™ scores for overweight children were approximately 2 units of the measure lower (i.e., worse) than FMS™ scores for healthy weight children. This too represents a moderate-to-large effect size once standardised. Figure 5 suggests considerable heterogeneity in correlation coefficients between studies. There was an *I*^2^ value of 90% and a small-to-moderate sized *Tau* value of 0.29. However, despite such heterogeneity, Fig. 5 also shows that there was no heterogeneity whatsoever in the direction of the effect, i.e., all correlation coefficients were negative.

## Discussion

Functional movement proficiency represents an important building block for lifelong (and potentially even injury-free) engagement in organised sport and PA. The FMS™ is a leading assessment tool for evaluating functional movement; however, to date, research has been primarily focused on FMS™ in the context of athletic populations (with some research in adult populations also). Research is lacking on the extent to which primary and secondary school-level children and adolescents exhibit functional movement proficiency (in particular, as measured by prominent assessment tools such as the FMS™).

This purpose of this study was to review and quantitatively synthesise published FMS™ data from such samples of children and adolescents, thereby providing normative reference values for PA specialists working in physical education and other non-competitive or non-elite forms of school sports and games. The study signals possible sex- and age-related differences in FMS™ scores. Particularly noteworthy is the degree of variability in FMS™ means between samples of secondary school-aged children. Variability in FMS™ means were five times greater in samples of secondary school-aged adolescents than it was in primary school-aged children (signalling potential developmental and maturation effects). Also of importance to this study was the degree to which the pooled data showed a moderate-to-large negative association between BMI and functional movement proficiency. While we were unable to provide an estimate of the direction of causality between BMI and FMS™, this study does consolidate the existing research evidence in that BMI and functional movement proficiency were interlinked. The substantive contributions of this study to the literature on functional movement, and FMS™ assessment in particular, are summarised in the following sections.

### Normative Scores for FMS™ Among Children and Adolescents

The establishment of a normative reference value for FMS™ in children and adolescents is an important outcome of this systematic review and meta-analysis. While normative values for adolescents were purported by Abraham et al. [44], this meta-analysis synthesises a far more comprehensive set of data from Europe, North America, the Middle East, and South Asia [44]. In other words, these data can be considered as a more comprehensive global normative for FMS™ scores among children and adolescents.

The meta-analytic mean score (14.06) reported in this study indicates that primary and secondary school-aged children exhibit potential deficits in functional movement and that they might be at risk for developing dysfunctional movement patterns over the course of an important period of maturation [2, 3]. Dysfunctional movements are characterised by a score of ‘1’ on any of the seven movements of the FMS™, and these movement patterns have been associated with injury risk and potentially damaging musculoskeletal health [18, 72]. Dysfunctional movements may contribute to low levels of mobility and poor levels of balance in performing motor tasks [18, 56, 58, 72]. This study signals a direct need for further assessment and monitoring of functional movement quality among typically developing groups of children and adolescents. The FMS™, for example, could be a viable addition to assessment within physical education.

### Sex Differences in FMS™

The current systematic review and meta-analysis also signalled potential sex differences in FMS™ means between samples of male and female school-aged children and adolescents. The data suggest that, on average, samples of females exhibit greater functional movement proficiency (a small but substantial effect magnitude of Cohen’s *d* =  − 0.27) when compared with their male counterparts. These findings compare well with the broader research evidence, which illustrates that females exhibit higher levels of mobility and flexibility over the lifespan [59–62]. However, the difference was not statistically significant, which means that there are possible interaction effects, possibly with age. For example, the age ranges of samples in this study might explain this residual uncertainty in the effect size representing sex differences in FMS™, particularly because a large number of studies included in this study spanned an age range of 9–14 years.

It is highly likely that differences in levels of maturation explain these findings. Across older samples, females are likely to have commenced puberty [63, 64]. In this context, females will experience and exhibit strength and neuromuscular gains associated with pubertal status, as well as their peak height velocity [59, 60]. In short, females and males in the age range for these samples naturally differ in flexibility, strength, and neuromuscular capacity, and this may be contributing to their FMS™ scores [60, 65]. In addition to this, at this age, males have higher levels of neuromuscular change during puberty, which can result in a period punctuated by lower motor control and imprecise bodily actions [65, 66]. It should be noted that, similar to FMS™ studies among older athletic populations, these sex differences represent small (but still substantial) differences in functional movement performances [67].

Another plausible explanation for this residual uncertainty in the effect size representing sex differences in FMS™ is given by close examination of the Ghasempoor et al., and Abraham et al., studies [40, 44]. These studies are from Iran and India and are the only studies that provide data on FMS™ outside of Europe and North America [40, 44]. This begs the question of whether there are potential cultural differences in the assessment of functional movement, which has been observed for PA assessments and other movement skills [68, 69]. For example, more conservative attitudes to female PA have been documented in studies from South Asia and the Middle East, with women and girls in these regions typically achieving lower levels of PA than their female reference group in Europe [68, 70–72]. Cultural differences in South-Asia and the Arabian gulf may also contribute to these discussions. Within these cultures, PA participation for females can be discouraged and may not be prioritised in the same manner as it is for males [73, 74]. Taken together, it is possible that lower levels of PA and time afforded to skill development among young females in these specific regions could have resulted in males exhibiting higher FMS™ scores. The data used in this systematic review and meta-analysis prohibit us from being definitive in our interpretation. There is however a fruitful line of transnational FMS™ research on the extent to which functional movement proficiency is affected by intersectional influences, such as sex differences that can be linked directly to cultural expectations and norms. It should also be noted that countries within the Arabian Gulf have documented high levels of overweight and obesity in children and youth, which may contribute to lower levels of PA and negatively impact on FMS™ scores [75, 76]. Female body composition values within the Arabian Gulf are notably high, with previous evidence reporting that over 30% of adolescent females are overweight or obese [77]. As females have lower documented levels of PA when compared with their male counterparts [78] within the Arabian Gulf, these findings may also contribute to the existing sex disparity in FMS™, as reported in the current meta-analytic mean.

### School Level and FMS™

A particularly novel outcome of our meta-analysis is the extent to which we were able to show differences in FMS™ variability between samples of primary and secondary school-aged participants. Variability in FMS™ mean was approximately five times greater in the samples of secondary school adolescents. This finding is particularly noteworthy, as movement proficiency has often been theorised to improve with early aging and maturation [58]. Studies during childhood show that FMS™ scores increase with age [5, 53]. As with sex differences, it is possible that maturation could be impacting FMS™ performance in secondary school. It is plausible that, in the samples of secondary-school adolescents, participants were at the onset of, or different stages of, their pubertal journey [63, 64]. As adolescents experience substantial physical and hormonal changes, particularly changes in limb length, body mass and composition, and neuromuscular control, they could consequently lack the bodily competence and coordination to negotiate the demands of FMS™ assessment [79]. Comparatively, primary school children are far less likely to be undergoing such intense physiological and hormonal changes and this means that FMS™ performance in this population group will tend towards greater homogeneity [63, 64]. It is possible that issues such as sporting experiences and prior injuries may also impact on FMS™ performance at different ages [11, 80]. However, the data in support of these arguments were not reported in the current systematic review and meta-analyses, given that they do not fit the classification of typically developing children and adolescents.

### Body Mass Index and FMS™

The final major finding of this systematic review and meta-analysis is the degree to which we found a negative and moderate-to-large association between BMI and FMS™. In the literature, weight status has been shown to consistently impact functional movement performances during childhood and adolescence, with similar evidence found in late athletically determined adolescent populations [81, 82]. However, BMI is not without criticism, and, in the context of the data presented in this systematic review and meta-analysis, some caveats may be worth noting. BMI does not distinguish between mass and fat-free mass [83]. This differentiation is particularly notable, given that many participants across these studies have begun puberty, a period punctuated by greater fat-free mass accumulation in males and fat mass accumulation in females, with much of this build-up occurring in a temporary or fluctuating capacity [84, 85].

The meta-analysis of the association between BMI and FMS™ in this study provides a clear corroborating quantitative estimate of this effect, which has mostly been shown in smaller, disparate samples. Further evidence of a significant association between BMI and FMS™ scores have been found in adults, suggesting that high BMI may negatively impede functional movement abilities across the lifespan [9]. Children and youth who are overweight or obese have previously been cited as possessing lower levels of postural stability and control, in addition to inconsistent abilities to absorb power at the key joints (knee, hip, and ankle) [38, 86, 87]. As these elements are critical to refined functional movement performances, poorer scores in FMS™ among those who present with overweight or obesity appears likely. It has also been theorised that children and youth who are overweight or obese are susceptible to greater musculoskeletal disorders, which may reduce their motivation and inclination for participation in PA [88]. As BMI does not distinguish between mass (i.e., fat mass, fat-free mass or muscle mass), it is also possible that some participants in this study may have accumulated more muscle mass, which can result in reduced mobility and flexibility performances in specific tasks associated with the FMS™ [61, 62].

The development of strong functional movement principles, such as mobility and stability, could prove highly effective in efforts to maintain weight status, given the previously established associations between BMI and stability [89]. The influence of mobility and stability on numerous elements of locomotor and stability skills suggests that improving these concepts could not only improve FMS™ scores, and possibly weight status, but also that these may correspond to improvements in other non-sporting domains, such as running and jumping [19, 90]. However, the direction of causality between BMI and FMS™ remains an open question. It is possible that higher levels of BMI deter children and adolescents from regular engagement in PA, thereby resulting in fewer opportunities to development functional movement proficiency during a critical developmental period [91]. These findings are concerning, given that high levels of BMI are often associated with lower levels of motor skills and lower PA. High BMI, low FMS™ scores and reduced movement abilities could all contribute to reduced levels of PA and physical fitness [92–94], alongside an increased health risk.

## Study Strengths, Limitations and Future Research Directions

This is the first systematic review and meta-analysis to consolidate data from studies of FMS™ in school-aged children and adolescents. The study was conducted and reported in accordance with the PRISMA statement and provides a comprehensive risk-of-bias assessment that supports the strengthening of quality in future FMS™ research designs. In addition to providing normative reference values for FMS™ scores and variances for this population group, this study also provided estimates of differences in FMS™ means between primary and secondary school-aged boys and girls. As such, the findings presented in this study directly identify population groups from whom FMS™ interventions can be prioritised in future research, namely secondary school-aged boys and girls classified as overweight and obese. Despite the strengths of this research study and its substantive contribution to synthesising a relatively new strand of movement competency research, there are a number of limitations that should be noted and can provide stimulus for the design of new research studies.

First, our estimation of initial norms for FMS™ values in this population group is based on a relatively small subsample of *published* international data (19 studies of 4911 subjects), relative, that is, to sample sizes from studies of other movement competence assessments, such as the assessment of fundamental movement skills. The data synthesised in the present study can and should be complemented and extended by new international population-level studies of FMS™ among children and adolescents. This will increase the generalisability of the present study’s findings and further refine our ability to monitor population-level functional movement performances among children and adolescents.

Second, while we were successfully able to estimate differences in FMS™ means between primary and secondary school-aged children and adolescents, which serves as a good proxy for differences in FMS™ at different ages and stages of development, the available research evidence was almost exclusively cross-sectional. Future research about the developmental trajectory of FMS™ requires longitudinal research designs, or, at the very least, access to individual, rather than study-level, cross-sectional data so that the association between age and FMS™ scores can be more precisely estimated.

A final limitation of our systematic review and meta-analysis is the extent to which we were unable to estimate and comment on the relative merits of different types of interventions aimed at improving FMS™ scores. This is a limitation because our meta-analysis of cross-sectional studies clearly indicates that certain population groups represent viable targets for interventions to improve FMS™ scores (namely secondary school-aged boys and girls classified as overweight and obese). Of the four intervention-based studies incorporated into this systematic review and meta-analysis, only one was adequately designed and powered to detect meaningful or beneficial effects [45].

In the discussion outlined above, suggestions for new lines of research have been made. Greater focus on the biological and maturational mechanisms that explain sex- and age-related differences in FMS™ is warranted. Future research utilising more stringent and accurate measures of mass (e.g. Dexa scan, waist-to-hip circumference) are required to further evaluate the role of overweight and obesity on functional movement capacity. Additionally, more in-depth reporting on the status (i.e., trained in FMS™, certified in FMS™ or untrained) of those administrating FMS™ assessments are necessary to improve the quality of functional movement research. The associated methods for scoring FMS™ data (i.e., live-scoring functional movement on the day of data collection, when compared with retrospective video scoring at a later stage in university laboratories) should also be detailed within the empirical literature to improve researcher and practitioner assessment accountability. More challenging but equally important lines of research are also required. There is an open question about the influence of cultural expectations and norms on sex differences in FMS™ performance, for example. There is also scope for functional movement researchers to now prioritise longitudinal research data collection and to incorporate novel cross-lagged panel models, which would enable new answers to open up the direction of the associations between FMS™ performance, BMI and the regularity of engagement in PA. Future research on the types of activities that children and adolescents can engage in to improve FMS™ scores ought to be considered alongside such cross-sectional, longitudinal and transnational analysis. Early evidence, for example, suggests that resistance training interventions have been shown to benefit FMS™ performance [43, 50, 95]. The degree to which changes in body mass and differences in PA level mediate the effectiveness of such interventions also represents an important design for future consideration (i.e., to tease out the extent to which simple changes in body mass and small differences in PA can lead to improvements in FMS™ scores rather than any single activity).

## Declarations

### Availability of data and material

All data generated or analysed during the study are included in this published article and its supplementary information files.
